# The effect of remote ischemic preconditioning on upper extremity strength in healthy male adults: an updated systematic review and meta-analysis

**DOI:** 10.1186/s13102-025-01494-8

**Published:** 2026-01-30

**Authors:** Jian Li, Ruolan Yang, Bo Zhang, Tianshuo Han, Jiashuang Jiang, Shiran Wang, Zhentian Wei, Qi Han

**Affiliations:** 1 Key Laboratory of Sport Training of General Administration of Sport of China, International Joint Laboratory on High Performance Sports Research, Beijing Sport University, No. 48, Xinxi Road, Beijing, 100084 China; 2 Sports Nutrition Center, National Institute of Sports Medicine, No. 2, Sports Stadium Road, Beijing, 100763 China

**Keywords:** Remote ischemic preconditioning, Upper extremity strength, Meta-analysis, Healthy male adults, Muscle performance

## Abstract

**Background:**

Remote ischemic preconditioning (RIPC) is a non-invasive intervention involving brief, repeated limb ischemia and is suggested to enhance muscle performance. However, findings in healthy men remain inconsistent, and few studies have examined upper-limb strength. This meta-analysis provides the first quantitative synthesis of acute RIPC effects on upper-body strength in healthy male adults, aiming to clarify its efficacy and practical relevance.

**Methods:**

English (PubMed, Web of Science, Embase, Cochrane Library) and Chinese (CNKI, Wanfang, VIP, CBM) databases were systematically searched up to March 16, 2025. Randomized crossover trials examining acute RIPC effects on upper-limb strength in healthy male adults were included. Primary outcomes involved maximal, explosive, and endurance strength measures. The review was registered with PROSPERO (CRD420251184757). Data were pooled using random-effects models, and heterogeneity was assessed via the I² statistic. Prespecified subgroup analyses examined moderators such as RIPC protocol (cycle number/duration) and strength outcome type (maximal, explosive, endurance).

**Results:**

Six randomized crossover trials involving 84 participants met inclusion criteria. The meta-analysis showed a small, positive, but non-significant overall effect of RIPC on upper-limb strength (SMD = 0.24, 95% CI: -0.02 to 0.51, *p* = 0.07) with low-to-moderate heterogeneity (I² = 32%). Subgroup analyses revealed no significant effects for maximal (SMD = 0.03, *p* = 0.85) or explosive strength (SMD = 0.13, *p* = 0.75). A moderate but non-significant improvement was observed for strength endurance (SMD = 0.54, 95% CI: -0.01 to 1.10, *p* = 0.054) with moderate heterogeneity (I² = 60%). Neither the number of ischemia–reperfusion cycles (*p* = 0.40 for subgroup difference) nor the test time window (< 30 min vs. ≥ 30 min, *p* = 0.37) significantly moderated the effects.

**Conclusion:**

This first meta-analysis on RIPC and upper-limb strength suggests that acute RIPC does not reliably improve upper-body strength in healthy male adults. Despite a moderate effect size observed for strength endurance, the lack of statistical significance and the 95% confidence interval crossing zero indicate considerable uncertainty in this finding. Together with the limited and heterogeneous evidence, this indicates that current protocols do not consistently enhance strength performance. RIPC should therefore be regarded as a theoretically promising but practically unverified approach. Larger, well-controlled trials are needed to determine whether specific RIPC parameters or time windows can produce reproducible performance gains.

**Supplementary Information:**

The online version contains supplementary material available at 10.1186/s13102-025-01494-8.

## Introduction

Remote Ischemic Preconditioning (RIPC) is an intervention strategy that induces ischemia-reperfusion by transiently blocking partial distal blood flow to a limb, thereby enhancing the tolerance of the target [1]. It is a non-invasive intervention that involves a transient ischemia-reperfusion cycle of the limb and is usually achieved using a blood pressure cuff to add pressure to the limb. Since Murry et al. firstly reported the protective effects of ischemic preconditioning on the myocardium in 1986 [2], RIPC has gradually become significant area of interest in the field of sports medicine and rehabilitation due to its non-invasive nature and potential clinical value. In recent years, researchers have begun to focus on the effects of RIPC on exercise performance in healthy populations, especially its modulation of muscle strength, endurance, and recovery [3–5], and the underlying mechanisms may involve the release of humoral factors, such as adenosine and nitric oxide, which may improve oxygen utilization and reduce oxidative stress in skeletal muscle [6]. However, the results of existing studies are significantly divergent and systematic evaluations targeting upper limb strength are still scarce. In this paper, we integrate current evidence by using meta-analysis method to explore the effects of RIPC on upper limb strength and its underlying mechanisms in healthy male adults.

The protective effects of RIPC may be achieved through multiple pathways, including adenosine receptor activation [7], enhancement of the nitric oxide (NO) signaling pathway [8], optimization of mitochondrial function [9], and elevated resistance to oxidative stress [10]. Clinical studies have shown that RIPC reduces the risk of myocardial injury [11] and acute kidney injury in cardiac surgery patients [12]. These findings suggest that RIPC is not only indicated for pathological conditions, but may also enhance physiological functions in healthy individuals by regulating metabolism and hemodynamics.

The potential benefits of RIPC in building muscle strength are more controversial, especially in terms of its application to healthy populations. Upper limb strength is an important component of physical functioning, affecting both athletic performance and daily activities. While resistance training remains the primary method for improving muscular strength, there is growing interest in complementary strategies, such as RIPC, that may enhance the effectiveness of training or provide acute performance benefits [13]. Within this research scope, there is currently no consensus on the effects of RIPC on muscle performance. Several studies have investigated the effects of RIPC on muscle strength in healthy adults, but results have been mixed and there have been limited experiments on upper limb strength. Some studies have shown that RIPC significantly improved lower limb explosive strength (e.g., vertical jump height) [14] and endurance exercise performance (e.g., ride-to-exhaustion time) [15], and in addition, some studies have reported significant improvements in grip strength and isokinetic torque, the mechanisms of which may be related to improved efficiency of muscular oxygen utilization and delayed lactic acid buildup [16, 17]. However, other studies did not observe significant improvements in grip strength or upper-body power output with RIPC [18, 19], and some other studies did not find significant effects [20, 21]. These differences may be attributed to differences in RIPC protocols (e.g., number of ischemic cycles, cuff pressure, and number of cycles), differences in subject characteristics (e.g., training level), and outcome metrics [5, 22].

The physiological mechanisms underlying the potential benefits of RIPC on muscle performance are multifaceted. Studies have shown that RIPC can be used as a method to improve exercise performance, particularly for exercises that are dominated by the aerobic system and glycolytic energy supply [23], and that RIPC may enhance muscle oxygenation, reduce fatigue, and increase metabolic efficiency during exercise [24, 25]. In addition, RIPC has been shown to increase muscle reperfusion and power output during dynamic exercise [26]. However, the extent to which these mechanisms translate into improvements in upper limb strength remains unclear, particularly among healthy male adults, a population selected primarily to reduce heterogeneity by controlling for potential sex-specific confounders, such as the influence of the menstrual cycle on physiological responses [27, 28], which is why this population was selected for this study.

Upper extremity strength is a core indicator of some daily functional physical activities and certain athletic performance, but its response to RIPC may differ from the lower extremity. For example, the distribution of fiber types and hemodynamic characteristics of upper limb muscles (e.g., biceps brachii) are different from those of the lower limb [29, 30], and RIPC interventions mostly act on the lower limbs (e.g., thighs), which may remotely modulate upper limb function through neurohumoral pathways [31]. Although some studies have been conducted to investigate the effects of RIPC on upper limb strength, the sample sizes are small (ranging 10–30) and the results are inconsistent, as well as lacking a systematic summary.

To date, no meta-analysis has specifically evaluated the effects of RIPC on upper extremity strength in healthy male adults. This specific population has received particular attention due to its importance in sports and occupational settings, as well as the potential for rapid and quantifiable strength adaptations that people have demonstrated [32]. Therefore, the aim of this meta-analysis was to synthesize the existing evidence and quantitatively synthesize the effects of RIPC on upper limb strength for the first time for a group of healthy male adults through Meta-analysis to determine whether RIPC improves upper limb strength in healthy male adults and to explore potential modifiers of its effectiveness, exploring the moderating effects of the compression cycle and the time window of the test, with the results to provide a theoretical basis for athletic training, sports medicine, rehabilitation, and physical fitness level optimization.

## Methods

### Literature search strategies

This systematic review and meta-analysis was conducted in accordance with the Preferred Reporting Items for Systematic Reviews and Meta-Analyses (PRISMA) guidelines. The study protocol was registered on the International Prospective Register of Systematic Reviews (PROSPERO) (Registration number: CRD420251184757).

Potential studies were identified using four English databases, Web of Science, PubMed, Cochrance Library and Embase, and four Chinese databases, CNKI, Wanfang, Wipu and CBM. For the study of the effect of RIPC on upper limb muscle strength, the subjects were categorized into three groups: RIPC, muscle strength, and upper limb. For these three groups of search terms, relevant predefined database-specific terms were added to broaden the search. For each database, the date range was limited to January 1, 1985 (since RIPC was first discovered in 1986) to March 16, 2025. Searches were performed on the major databases including, but not limited to, “ischemic preconditioning”, “Preconditioning, Ischemic”, “Ischemic Pre-Conditioning”, “Ischemic Pre Conditioning”, “Pre-Conditioning, Ischemic”, “Muscle Strength”, “Strength, Muscle”, “Arthrogenic Muscle Inhibition”, “Arthrogenic Muscle Inhibitions”, “Inhibition, Arthrogenic Muscle”, “Muscle Inhibition, Arthrogenic”, “Upper Extremity”, “Extremities, Upper”, “Upper Extremities”, “Extremity, Upper”, “Membrum superius”, “Upper Limb”, “Limbs, Upper”, “Limb, Upper”, and “Upper Limbs” for subject + free word search.

To ensure high sensitivity, the search was not limited by the ‘Remote’ modifier. Studies involving local ischemic preconditioning were excluded during the screening process, as per our eligibility criteria.

### Study inclusion and exclusion criteria

The inclusion criteria for this study were as follows: (1) participants were healthy young men aged 18–39 years with no significant differences in baseline characteristics; (2) RIPC compression was applied to the upper limbs, with compression of 150 mmHg or higher, and the RIPC intervention cycle was three cycles or more; (3) the experimental group underwent RIPC intervention without any other interventions; (4) inclusion criteria were based on muscle strength categorized by functional performance: maximum strength, rapid strength, or strength endurance. Additionally, the study should provide sample size, mean, and standard deviation.

Literature will be excluded if one of the following occurs: (1) duplicate literature; (2) copyright issues preventing access to the full text; (3) studies in animals or non-healthy subjects; (4) pressurization locations other than the upper extremities; (5) papers with ambiguous data and incomplete outcome data; and (6) errors in the study design.

### Data extraction

Two authors (R.Y., and J.J.) independently extracted the data into pre-designed spreadsheets that included the following information: title, authors, year, experimental methodology, sample size, specific pressures of the intervention and controls, intervals between the intervention and the test, outcome metrics (1RM values, barbell power and duration of compression in the bench press exercise, number of repetitions and total workout in the bench press and shoulder press, and time to exhaustion), and outcomes (sample size, mean, and standard deviation). The outcome data were categorized for three dimensions of strength qualities: (1) maximal strength: 1RM value (bench press/anterior latissimus dorsi pulldown/shoulder press); (2) explosive strength: barbell power (peak/mean); and (3) strength endurance: number of repetitions, total training volume, compression time, and force exhaustion time. Meanwhile, two moderating variables were set according to the characteristics of the literature, which were the number of pressurization cycles (3 × 5min vs. 4 × 5min) and the testing time window (less than 30min vs. not less than 30min). The 30-minute cut-off was chosen a priori based on the physiological rationale of the ‘early window’ of RIPC protection, which is mediated by acute humoral and neural pathways and typically manifests within minutes to a few hours after the intervention [10, 33].

Since Novaes et al. (2021) did not report his research results in full, an attempt was made to contact the corresponding author to obtain the missing data, but no response was received. Consequently, data were extracted from Figs. 2 and 3 of Novaes et al. (2021) using WebPlotDigitizer software (GetData Graph Digitizer, version 2.26).

## Results

### Study selection

**Fig. 1:**
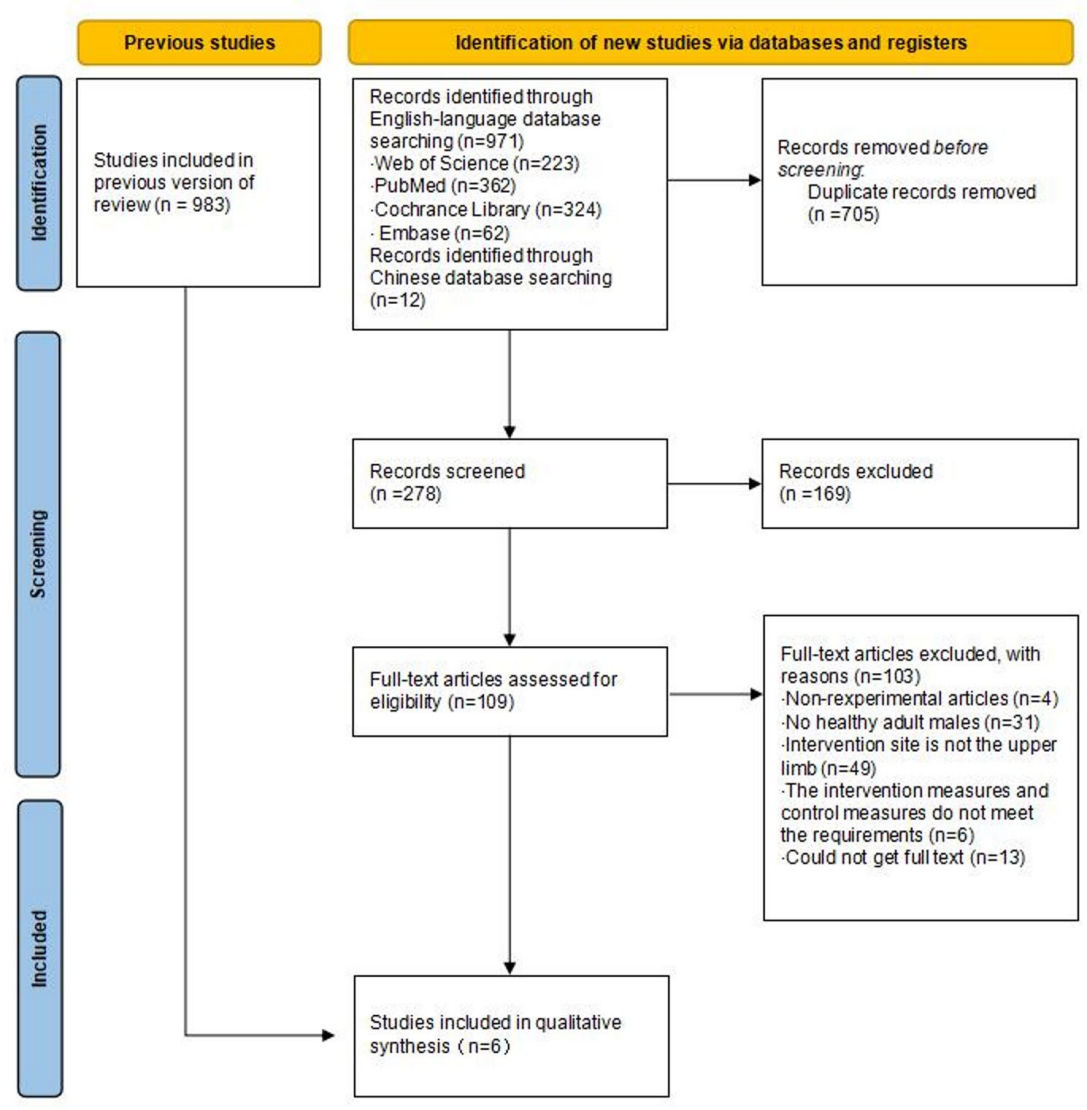
Literature search and study selection from PRISMA statement

A flowchart of the different stages of reviewing the literature according to PRISMA as shown in Fig. 1. In this paper, 983 studies related to RIPC, upper limb, and muscle strength were retrieved from different databases (Web of Science: *n* = 223, PubMed: *n* = 362, Cochrance Library: *n* = 324, Embase: *n* = 62, and Chinese databases: *n* = 12). Using Zotero literature management software, 705 duplicate studies were identified and removed. Subsequently, 169 studies were excluded based on title and abstract. Finally, full-text screening based on literature inclusion criteria further excluded 103 studies (1. Non-experimental articles: *n* = 4; 2. Non-healthy male adults: *n* = 31; 3. Intervention location non-upper limb: *n* = 49; 4. Interventions and controls not eligible: *n* = 6; 5. Full-text unavailable = 13). Therefore, a total of six studies were included.

### Characteristics of included studies

A total of six papers (2021–2024) were included in this study, all of which were randomized crossover trials. As shown in Table 1, only healthy male adults were included in a total of 84 subjects with an average age ranging from 20.34 ± 1.70 to 29.9 ± 5.9 years old. In terms of RIPC intervention, ischemic pressure was 170 mmHg in two studies and 220 mmHg in four; the number of cycles was 3 × 5 min in three studies and 4 × 5 min in three; and the test window period was less than 30 min in four studies and not less than 30 min in two. The findings of three out of six studies yielded a significant positive effect: The study by Novaes et al. (2021) noted that RIPC significantly increased the number of repetitions and total workload with localized and distal effects [34]; Rodrigues et al. (2023) concluded that IPC significantly increased the 1-RM and decreased subjective fatigue [35]; and Telles et al. (2022) reported that both high and low intensity RIPC acute intervention elevated maximal strength [36]. Three studies concluded no significant effect: Valenzuela et al. (2021) found that RIPC did not benefit the bench press load-velocity relationship, 1-RM, and number of reps to exhaustion [37]; Niu et al. (2024) concluded that RIPC did not improve the performance of bodybuilders in the exhaustion bench press [38]; and Bellini et al. (2023) reported that RIPC increased the duration of time to forced exhaustion but did not alter oxygen uptake kinetics [39].

**Table 1:** Characteristics of included studies

Authors(year)	Country	Subjects	Age(years)	Ischemia pressure (mmHg)	IPC sets	Intervention site	Time to test	Outcome measurement	Findings
Pedro L. Valenzuela(2021)	Spain	16 males	23.0 ± 2.0	220	3 × 5 min	Both arms	40 min	Bench press 1-RM, The number of repetitions for the bench press at 60% 1-RM	Bilateral RIPC applied on the upper limbs 40 min before exercise provided no acute benefits on the load velocity relationship, maximal strength (i.e., 1RM), or the number of repetitions that can be performed to failure in the bench press exercise
Xuehan Niu(2024)	China	10 male bodybuilders	20.34 ± 1.70	170	3 × 5 min	The upper arm close to the proximal end, alternating between upper limbs	30 min	Peak power (PP), mean power (MP), time under tension (TUT)	The triple-cycle RIPC intervention does not improve the upper limb strength performance of bodybuilding athletes in exhaustive bench presses.
Jefferson da Silva Novaes(2021)	Brazil	16 recreationally trained and normotensive men	24.8 ± 2.2	220	4 × 5 min	Around the subaxillary region of the upper arm, alternating arms	5 min	The number of repetitions for several actions, Total volume of work performed	RIPC significantly increased the number of repetitions and the total volume of work performed, compared with the CUFF and control protocols, in an RE training session with 6 multijoint exercises, which combined involved the major muscle groups. Importantly, IPC generated both local and remote beneficial effects without affecting perceived exertion during exercise
Anderson Luiz Rodrigues(2023)	Brazil	15 resistance-trained men	29.9 ± 5.9	170	3 × 5 min	At the proximal area of both arms	10 min	Bench press 1-RM	RIPC significantly improves bench press 1-RM load and reduces session-RPE in resistance-trained men. Such evidence suggests an ergogenic effect of RIPC with potential application in competitions or training sessions involving repeated maximal strength output.
D. Bellini(2023)	UK	11 recreationally-active males	24 ± 2	220	4 × 5 min	As proximal as possible on the upper arm	12 min	Time-to-exhaustion (TTE)	TTE was longer following RIPC during upper-body exercise despite unchanged V̇O2 kinetics.
Luiz Guilherme Telles(2022)	Brazil	16 recreationally trained men	25.3 ± 1.7	220	4 × 5 min	Around the subaxillary region of the upper arm	10 min	Bench press 1-RM, front latissimus pull-down 1-RM, shoulder press 1-RM	Both RIPC maneuvers with high and low pressure acutely increased the maximum strength in varied resistance exercises compared with the CON condition.

### Quality analysis

**Fig. 2:**
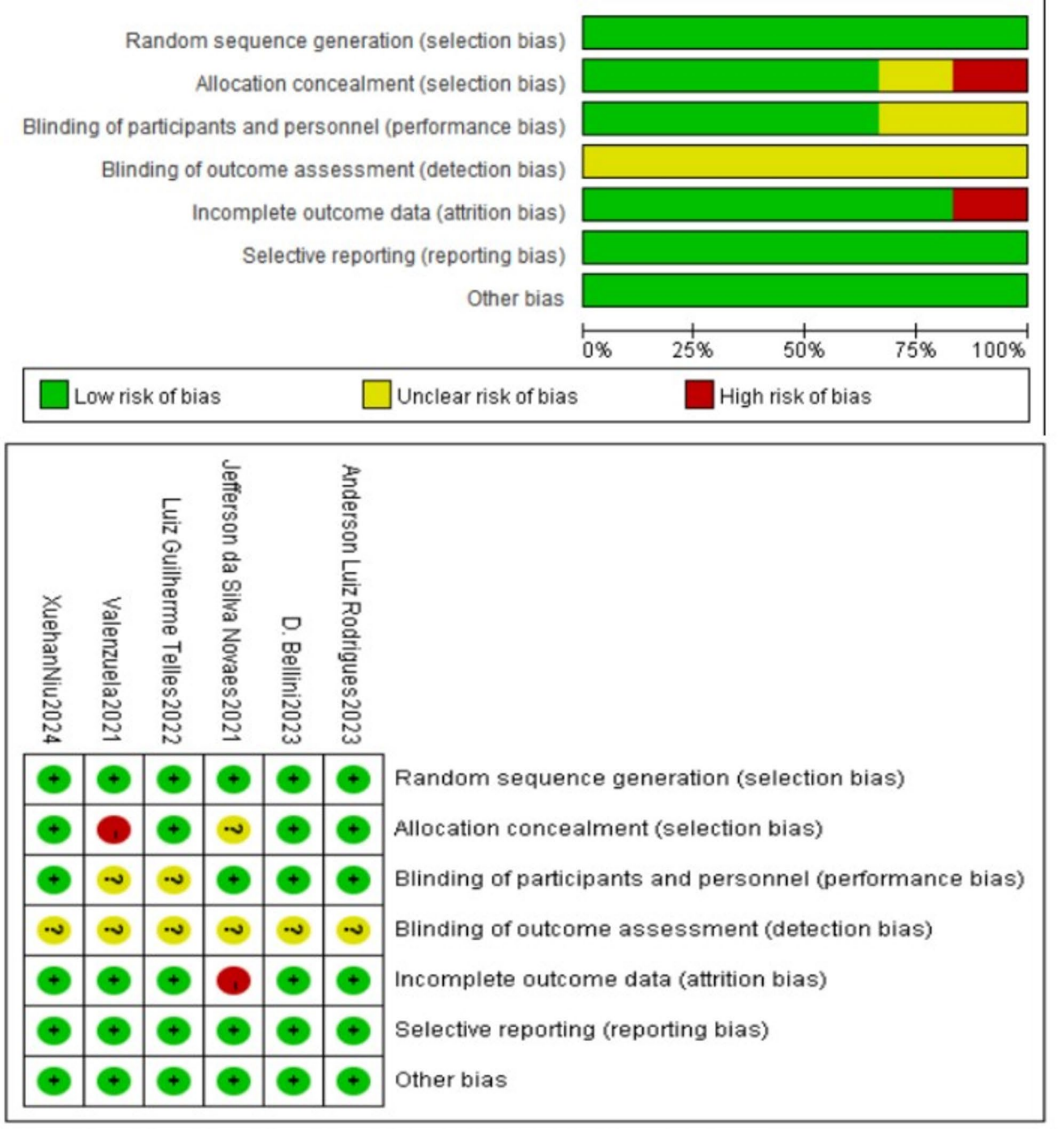
Risk of bias summary for included studies, assessed using the Cochrane Risk of Bias tool

The methodological quality of each independent study included was assessed using the Cochrane Risk of Bias Assessment Tool (RoB 2) (Fig. 2). Assessments included (1) generation of randomized sequences; (2) allocation concealment; (3) implementer-participant double-blinding; (4) blinding in outcome assessment; (5) data completeness; (6) selective reporting; and (7) other biases. Assessments were conducted independently by two researchers and results were reviewed jointly. If there were disagreements, they were discussed or the opinions of third-party researchers were consulted and analyzed after combining the opinions. Of the six articles included in this study, four (66.66%) were free of high-risk factors. The categories of randomized allocation scheme, selective reporting of study results, and other sources of bias were all at 100% low risk. A total of four articles (66.66%) were explicitly blinded. All six articles met the criteria for randomized sequence generation. None of the six articles explicitly reported blinding in the assessment of outcomes. Outcome data were not reported in full in one study (16.66%). All studies were free from selective reporting and other biases. In conclusion, the overall quality of studies included in this study was high.

### Overall effects analysis

#### Collective effect

**Fig. 3:**
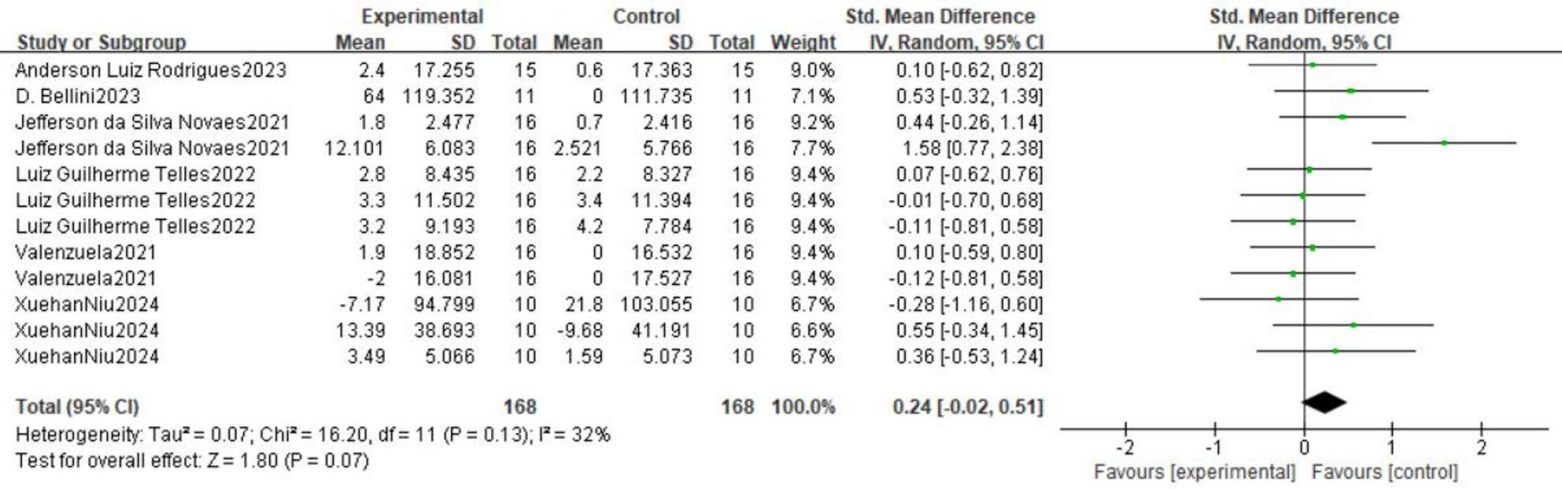
Forest plot for comprehensive analysis of included documents

**Fig. 4:**
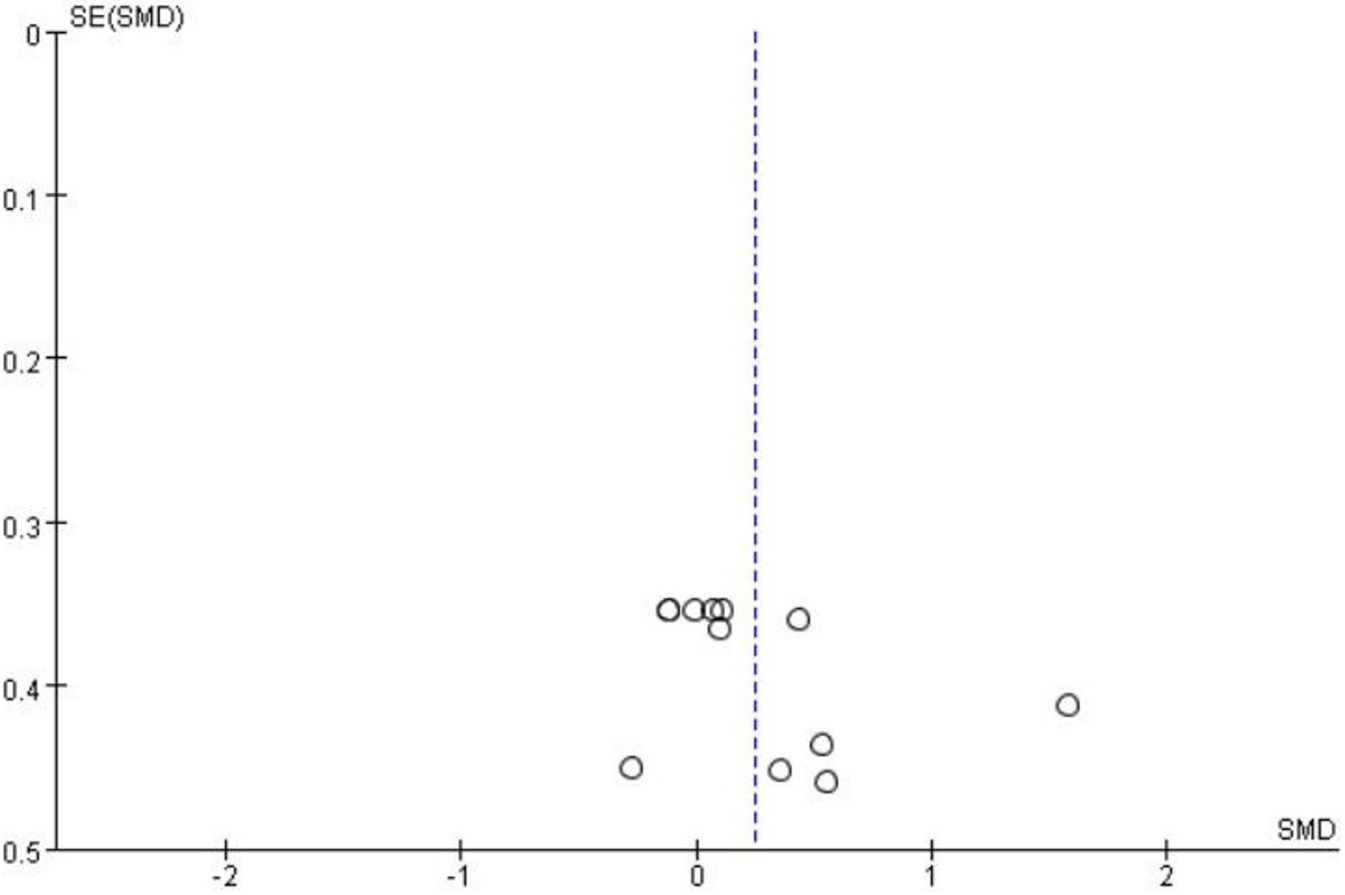
Funnel plot for comprehensive analysis of included documents

The forest plot (Fig. 3) presents the pooled results of all outcome data for the effect of remote ischemic preconditioning (RIPC) on upper limb strength. The random-effects model meta-analysis indicated that RIPC demonstrated a small, positive, but statistically non-significant overall effect on the upper limb strength of healthy male adults (SMD = 0.24, 95% CI: -0.02 to 0.51, *p* = 0.07). Heterogeneity among the included studies was low to moderate (I² = 32%, *p* = 0.13). Although the point estimate suggests a potential benefit of RIPC, the 95% confidence interval crossing the line of no effect (SMD = 0) indicates that the overall effect did not reach conventional statistical significance. The funnel plot (Fig. 4) appeared roughly symmetrical, suggesting a low risk of publication bias. The use of a random-effects model provides a more conservative and generalized estimate, accounting for potential methodological variations across studies.

#### Strength qualities

**Fig. 5:**
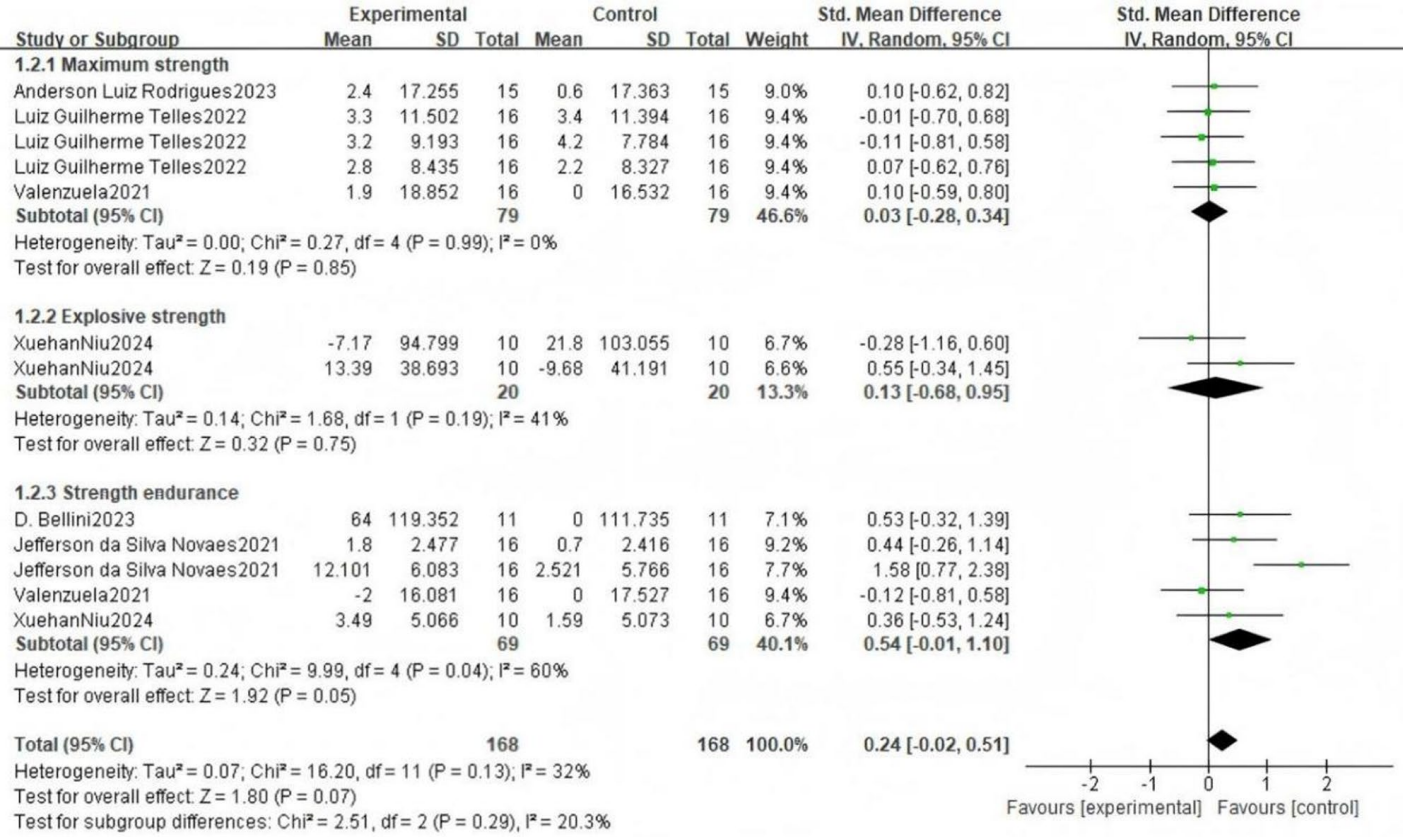
Forest plot including articles classified by strength quality

**Fig. 6:**
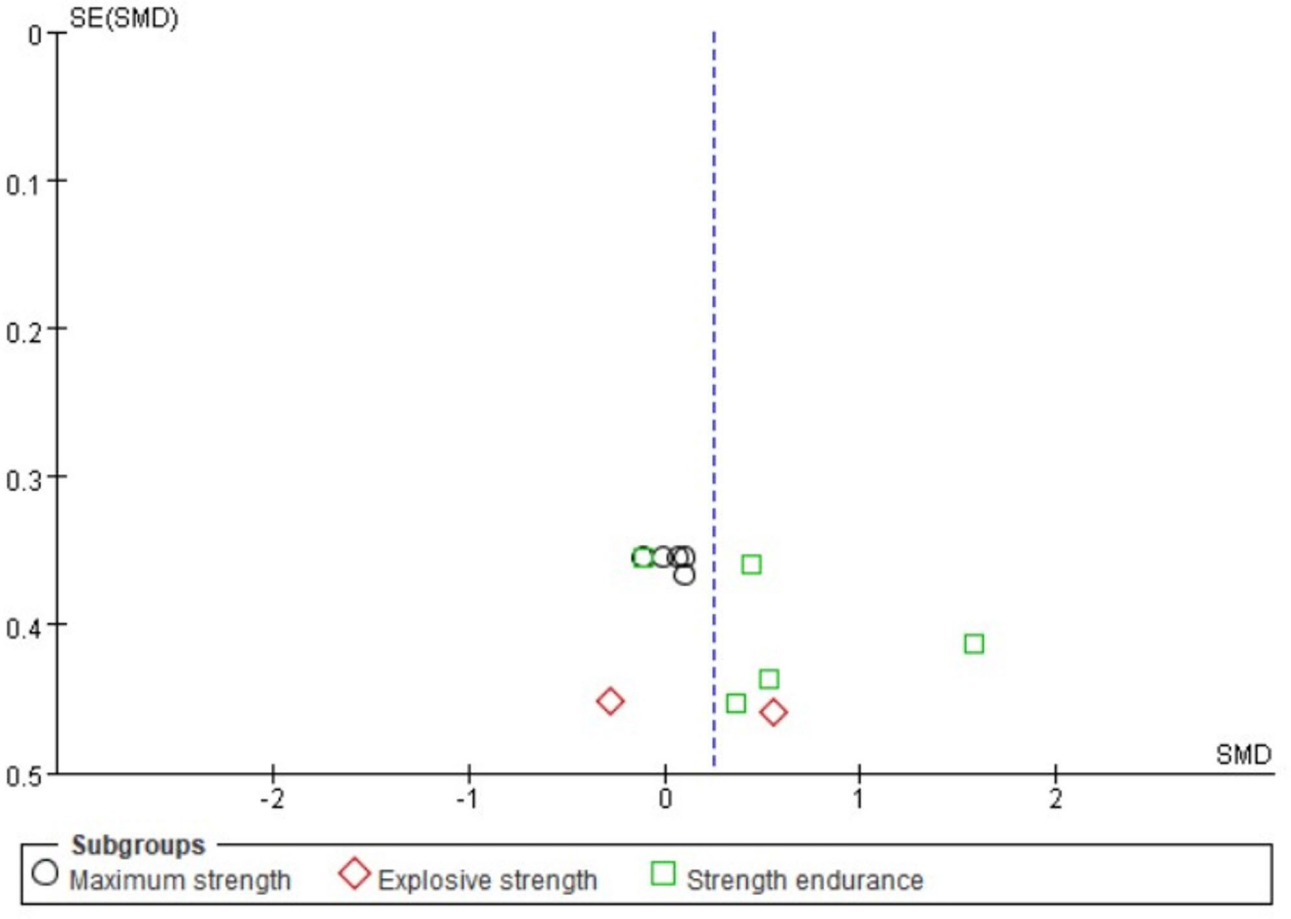
Funnel plot for included articles classified by Strength Quality

Subgroup analysis was performed based on the types of strength quality: maximal strength, explosive strength, and strength endurance (Fig. 5). For maximal strength, the pooled results from five measurements across three studies showed a negligible and non-significant effect (SMD = 0.03, 95% CI: -0.28 to 0.34, *p* = 0.85), with no heterogeneity observed (I² = 0%). Regarding explosive strength, the combined analysis of peak and mean power from one study revealed a small, non-significant positive effect (SMD = 0.13, 95% CI: -0.68 to 0.95, *p* = 0.75), with low heterogeneity (I² = 41%). In contrast, the analysis for strength endurance, which pooled data from five measurements across four studies, indicated a moderate positive effect (SMD = 0.54, 95% CI: -0.01 to 1.10). Given that the 95% confidence interval includes the null value and the conventional threshold for statistical significance is *p* < 0.05, we sought to verify the precise p-value. As the primary analysis in RevMan 5.4 reports p-values to two decimal places (*p* = 0.05), we conducted a supplementary analysis using R software (version 4.4.2) with the metafor package to obtain greater precision. This analysis yielded a more precise p-value of 0.054. Thus, this finding should be interpreted as non-significant given that the p-value exceeds the conventional threshold of 0.05. Furthermore, the 95% confidence interval crossing zero reinforces the uncertainty of this estimate. Therefore, the effect for strength endurance is appropriately interpreted as a non-significant, positive finding. This subgroup also exhibited substantial heterogeneity (I²= 60%). The test for subgroup differences did not reveal a statistically significant difference between the three strength qualities (*p* = 0.29). The funnel plot for this analysis (Fig. 6) showed a roughly symmetrical distribution of studies, suggesting a low risk of publication bias across the subgroups.

In summary, remote ischemic preconditioning did not demonstrate significant effects on maximal strength, explosive strength or strength endurance in healthy male adults. The positive effect size observed for strength endurance was not statistically significant and was characterized by substantial heterogeneity, indicating considerable uncertainty in this estimate.

### Moderator variable analysis

#### Effect of subgroups of the number of pressurization cycles (3×5min vs. 4×5min) on the three types of forces

**Fig. 7:**
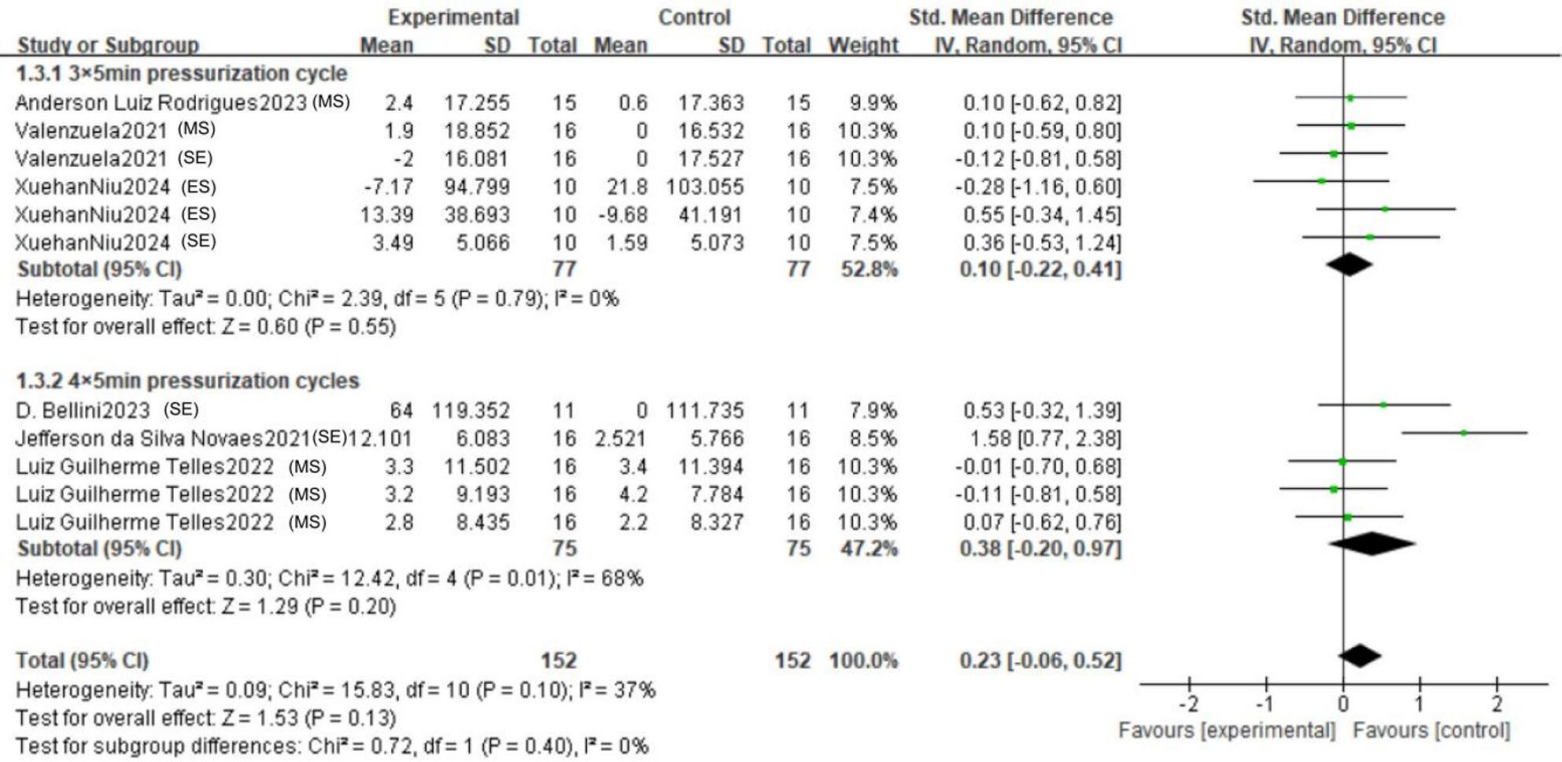
Forest plots with different numbers of pressure cycles (MS: Maximum strength; ES: Explosive strength; SE: Strength endurance)

To investigate whether the number of pressurization cycles serves as a potential moderator, we performed a subgroup analysis comparing the effects of the 3 × 5-minute and 4 × 5-minute protocols (Fig. 7). The pooled estimate for the 3 × 5-minute protocol indicated a negligible and non-significant effect on upper limb strength outcomes (SMD = 0.10, 95% CI: -0.22 to 0.41, *p* = 0.55). The absence of heterogeneity (I² = 0%) suggests a consistent lack of effect across the diverse strength qualities (maximal strength, explosive strength, and strength endurance) within this subgroup. Conversely, the 4 × 5-minute protocol yielded a higher, yet still non-significant, point estimate (SMD = 0.38, 95% CI: -0.20 to 0.97, *p* = 0.20). This analysis was, however, marked by substantial heterogeneity (I² = 68%), indicating considerable inconsistency in the results among the included studies. Critically, the test for subgroup differences confirmed that the disparity between the two protocols was not statistically significant (*p* = 0.40).

**Fig. 8:**
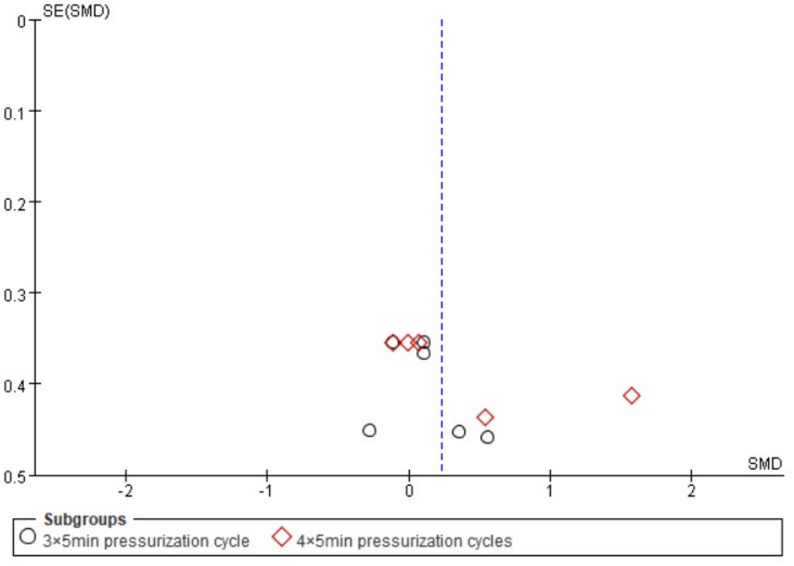
Funnel plot with different numbers of pressure cycles

A detailed examination of the forest plot elucidates these findings. Within the 3 × 5-minute subgroup, the confidence intervals for all individual studies encompass the null value, reinforcing the conclusion of no clear effect. For the 4 × 5-minute subgroup, the overall effect appears disproportionately influenced by the outcomes in the strength endurance domain. Specifically, while results for maximal strength are tightly clustered around zero, the two strength endurance studies show positive but inconsistent effects, with one (Novaes et al. 2021) demonstrating a large, significant effect and the other (Bellini et al. 2023) a modest, non-significant one. This stark contrast likely contributes to the high heterogeneity observed. The absence of data on explosive strength in the 4 × 5-minute subgroup limits a comprehensive assessment. The funnel plot (Fig. 8) exhibited approximate symmetry, implying a low likelihood that publication bias substantially impacted these subgroup comparisons.

In conclusion, based on the available evidence, the number of pressurization cycles does not demonstrate a statistically significant moderating effect on the efficacy of RIPC. The nominally larger effect size associated with the 4 × 5-minute protocol should not be overinterpreted, as it is statistically non-significant, driven by inconsistent findings within a single strength quality, and not significantly different from the 3 × 5-minute protocol. Future research with larger sample sizes and standardized outcome measures is warranted to definitively explore the potential dose-response relationship of RIPC.

#### Effect of test time window subgroups (less than 30 min vs. not less than 30 min) on three types of strengths

**Fig. 9:**
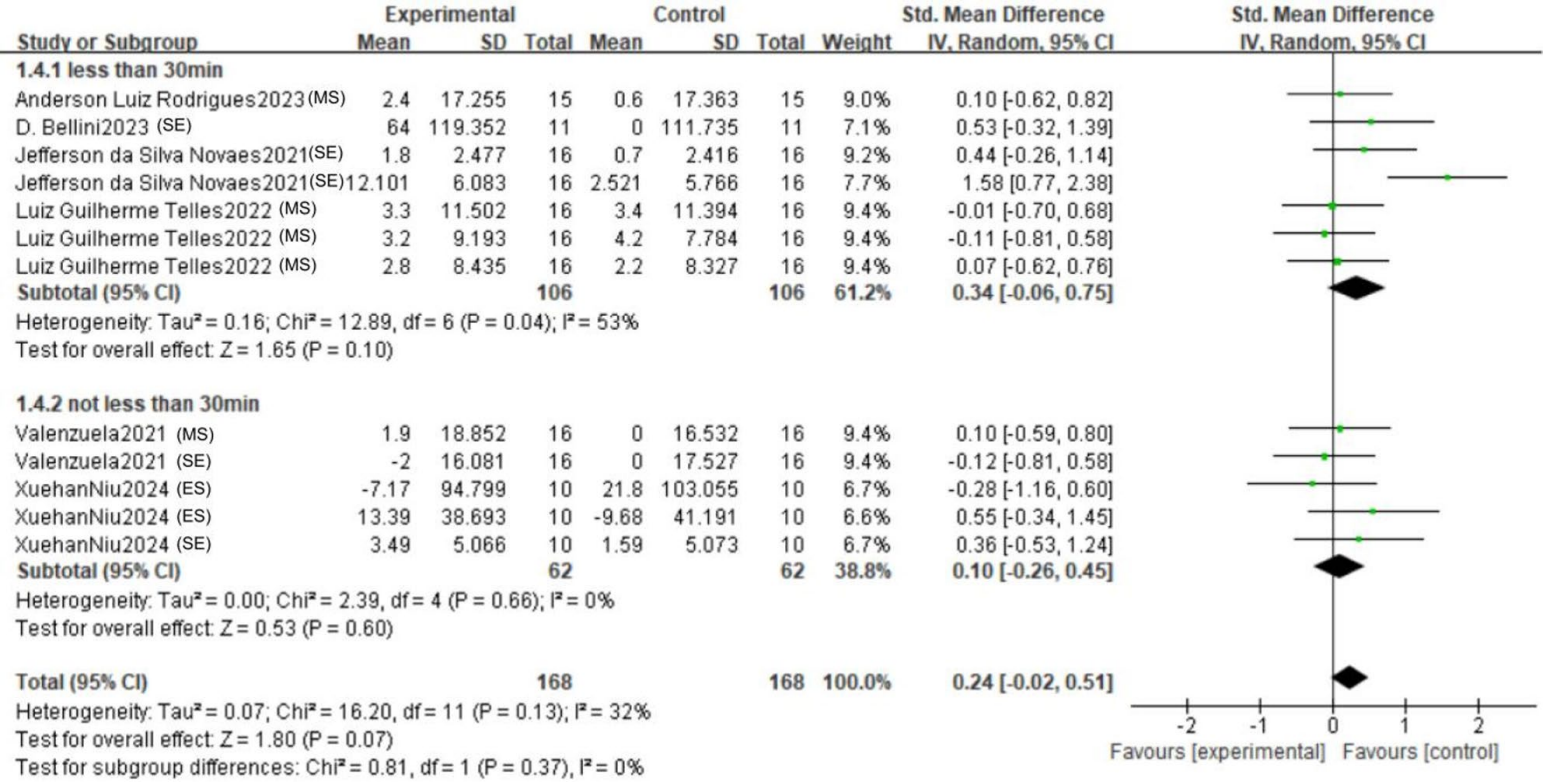
Forest plots with different numbers of pressure cycles (MS: Maximum strength; ES: Explosive strength; SE: Strength endurance)

To examine the potential influence of the time interval between the RIPC intervention and strength assessment, we conducted a subgroup analysis comparing effects measured within 30 min versus those measured at 30 min or later (Fig. 9). The overall pooled effect for studies with a test time window of less than 30 min was positive but not statistically significant (SMD = 0.34, 95% CI: -0.06 to 0.75, *p* = 0.10). This analysis was characterized by considerable heterogeneity (I² = 53%), indicating substantial inconsistency among the results in this subgroup. In contrast, the pooled effect for studies with a test time window not less than 30 min was negligible and non-significant (SMD = 0.10, 95% CI: -0.26 to 0.45, *p* = 0.60), with no observed heterogeneity (I² = 0%), suggesting consistent null findings across these studies. Critically, the test for subgroup differences revealed no statistically significant difference between the two time windows (*p* = 0.37).

**Fig. 10:**
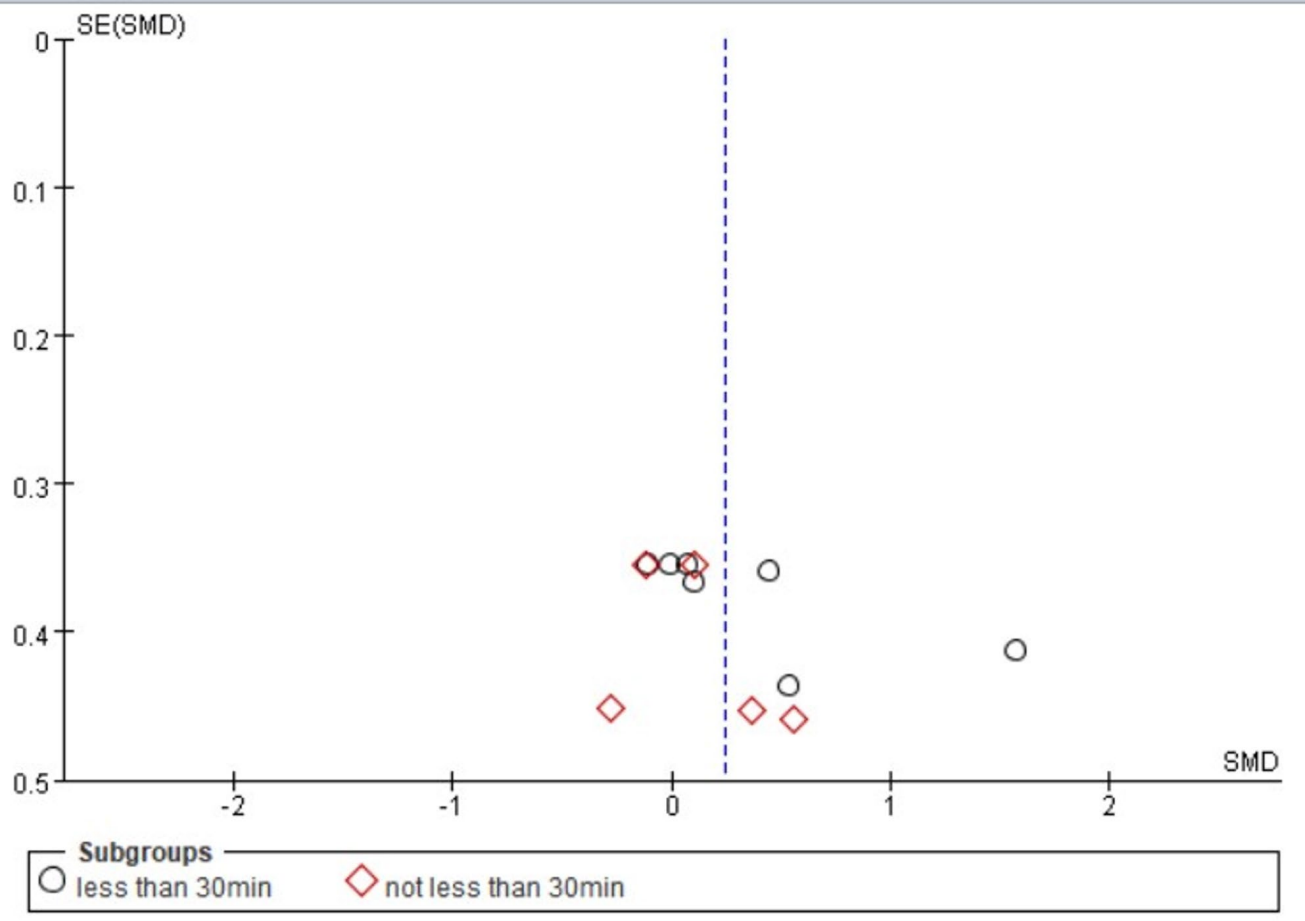
Funnel plots with different numbers of pressure cycles

An inspection of the forest plot reveals the source of heterogeneity in the “<30min” subgroup. While most individual study estimates are non-significant, one study on strength endurance (Novaes et al. 2021, SE) demonstrated a large and significant effect, which appears to be a primary driver of both the elevated point estimate and the statistical heterogeneity in this subgroup. The “≥30min” subgroup presents a more homogeneous picture, with all confidence intervals narrowly spanning the null value. The funnel plot (Fig. 10) exhibited a roughly symmetrical distribution of effect sizes around the overall pooled estimate, suggesting a low risk of publication bias influencing these subgroup comparisons.

In conclusion, the test time window (< 30 min vs. ≥30 min) was not identified as a statistically significant moderator of RIPC’s effect on upper limb strength. Although the point estimate was higher for the “<30min” subgroup, this finding was non-significant, statistically heterogeneous, and not significantly different from the “≥30min” subgroup. Therefore, based on the current body of evidence, the timing of post-intervention testing within the examined ranges does not appear to systematically alter the efficacy of RIPC. Future studies with standardized and multiple post-intervention testing time points are warranted to more definitively explore the temporal dynamics of the RIPC effect.

## Discussion

### Summary of key findings

This systematic review and meta-analysis quantitatively synthesized evidence from six randomized crossover trials to evaluate the efficacy of remote ischemic preconditioning (RIPC) on upper limb strength in healthy male adults. The primary finding is that RIPC demonstrated a small, positive, but statistically non-significant overall effect on composite measures of upper limb strength (SMD = 0.24, 95% CI: -0.02 to 0.51, *p* = 0.07). Subsequent subgroup analyses revealed dimension-specific effects. Specifically, RIPC did not produce significant improvements in either maximal strength (SMD = 0.03, *p* = 0.85) or explosive strength (SMD = 0.13, *p* = 0.75). In contrast, a moderate positive but non-significant effect was observed for strength endurance (SMD = 0.54, *p* = 0.054), and this analysis was marked by substantial heterogeneity (I² = 60%).

Furthermore, analyses of potential moderating variables indicated that neither the number of pressurization cycles (3 × 5 min vs. 4 × 5 min; subgroup difference *p* = 0.40) nor the testing time window (< 30 min vs. ≥30 min; subgroup difference *p* = 0.37) significantly influenced the overall results. The nominally larger effect size associated with the 4 × 5-minute protocol (SMD = 0.38) was not statistically significant (*p* = 0.20) and was highly heterogeneous. Similarly, the point estimate for the < 30-minute time window (SMD = 0.34) was also non-significant (*p* = 0.10) and heterogeneous. In both cases, the observed effects appeared to be disproportionately driven by positive outcomes from a limited number of studies within the strength endurance domain.

### Comparison with existing studies

The findings of this meta-analysis broadly mirror the mixed results reported in the literature. For example, studies by Novaes et al. (2021) and Rodrigues et al. (2023) showed clear performance benefits: Novaes et al. found that RIPC (4 × 5-min at 220 mmHg) significantly increased repetition count and total work volume in a multi-joint resistance training session [34], and Rodrigues et al. reported higher bench-press 1RM after RIPC [35]. Similarly, Telles et al. (2022) observed that both high- and low-pressure RIPC acutely elevated maximal strength in varied resistance exercises [36]. In contrast, Valenzuela et al. (2021) observed no improvement in bench-press force outcomes after RIPC [37], and Niu et al. (2024) reported that a 3 × 5-min RIPC intervention did not enhance exhaustive bench-press performance in trained bodybuilders [38]. Bellini et al. (2023) provide another nuanced result: RIPC significantly increased time-to-exhaustion during upper-body arm-crank tests but did not alter VO₂ kinetics [39]. In our meta-analysis, similarly, we found no significant effect of RIPC on maximal or explosive upper-limb strength, but a moderate but non-significant effect for strength endurance. This aligns with Bellini’s finding of improved endurance (longer exercise tolerance) despite unchanged metabolic markers. Overall, some studies report acute gains in strength or power, while others report null effects. These discrepancies likely stem from differences in study design. For instance, protocols varied in occlusion pressure (170 vs. 220 mmHg), number of cycles (3 × 5 min vs. 4 × 5 min), and timing of testing (e.g. Novaes et al. tested immediately after RIPC, Valenzuela et al. utilized a 40-minute rest interval). Subject characteristics also differed: many null-result studies used highly trained athletes (e.g. competitive bodybuilders), whereas positive studies often involved recreational exercisers. Outcome measures varied as well (e.g. 1RM vs. power or endurance tests). These methodological factors can influence results. In summary, our meta-analytic finding of a small, non-significant overall effect is consistent with several previous trials reporting no acute strength benefit (e.g. Valenzuela et al. 2021; Niu et al. 2024), while other studies’ positive results (Novaes et al. 2021; Bellini et al. 2023) appear to be largely confined to strength-endurance outcomes or specific protocols, it is critical to note that these individual findings were not corroborated by our pooled analysis, which showed non-significant effects when all evidence was considered.

### Explanation of physiological mechanism

Remote ischemic preconditioning (RIPC) is believed to enhance muscular performance via multiple interrelated mechanisms: humoral signalling, endothelial/microvascular improvements, mitochondrial and metabolic adaptations, and neural modulation of motor output and afferent feedback. First, brief RIPC cycles applied to a remote limb release circulating mediators such as adenosine, bradykinin and endogenous opioids, which act systemically to confer protective and performance-enhancing effects [18, 33]. Enhanced muscle perfusion can improve O₂ and substrate delivery, accelerate metabolite clearance (e.g., lactate, H⁺) and thus delay peripheral fatigue, a plausible underlying mechanism improved endurance outcomes reported in some RIPC studies [6, 40, 41].

Second, RIPC has been shown to impact mitochondrial function and bioenergetic efficiency. Pre-clinical and translational research indicates that ischemic preconditioning stimulates protective signalling (e.g., reperfusion injury salvage kinase, PKC pathways) and preserves mitochondrial integrity during subsequent metabolic stress [18, 33]. Preservation of mitochondrial function supports more efficient ATP resynthesis and reduces reactive oxygen species (ROS) accumulation, thereby benefiting tasks that depend on repeated contractions or sustained output rather than single maximal efforts [42].

Third, neural modulation appears to contribute. There is evidence that RIPC may attenuate group III/IV afferent feedback (which ordinarily inhibits central motor drive during fatigue) or modulate autonomic/motor neuron excitability, thus allowing more voluntary output or prolonged performance [10, 42]. This neural-feedback modulation may help explain why some studies reported improvements in repeated-effort or time-to-exhaustion tasks [41], whereas maximal voluntary strength (1RM) appears less consistently influenced by RIPC - it likely because 1RM is dominated by immediate neuromuscular activation, which RIPC influences only indirectly.

Fourth, metabolic and buffering effects have been noted. Several investigations reported that ischemic preconditioning modified lactate kinetics, reduced the decline in power output during repeated sprints, or altered substrate utilisation [40, 43]. For example, Cheng et al. (2021) found that remote ischemic preconditioning improved total work and reduced power decrement during sprint interval exercise in team-sport athletes [44]. However, whole-body VO₂peak is not consistently improved, suggesting that the major effects of RIPC may lie in peripheral muscle-level adaptations rather than central oxygen uptake improvements.

Finally, the temporal dimension matters: RIPC appears to produce an “early window” (minutes to a few hours) mediated by acute humoral and vascular responses, and a “late window” (≈ 24–72 h) dependent on gene transcription and protein synthesis [10, 33]. In exercise contexts, this means the time interval between RIPC and performance testing may critically affect which mechanisms predominate and hence which performance domains benefit.

In summary, mechanistic evidence supports the biological plausibility that RIPC might modestly enhance strength-endurance or repeated-effort performance by improving muscle perfusion, mitochondrial efficiency and attenuating fatigue-related afferent signaling. Conversely, maximal strength or explosive power, which are more dependent on instantaneous neuromuscular activation and less on perfusion/metabolic fatigue, appear less consistently influenced by RIPC. The multi-pathway nature of RIPC (humoral → vascular → metabolic → neural) implies that its functional translation will depend on the physiological demands of the task and the specifics of the RIPC protocol. However, it is important to note that while these mechanisms provide biological plausibility for potential endurance benefits, our meta-analytic findings do not provide conclusive evidence for such effects in the context of upper-limb strength endurance.

### Role of moderating variables

In the present meta-analysis, two commonly discussed moderators were analysed: the number of ischemic cycles (3 × 5 min vs. 4 × 5 min) and the time interval between RIPC application and performance testing (< 30 min vs. ≥ 30 min). Neither moderator reached statistical significance in our subgroup analyses (*p* = 0.40 for cycles; *p* = 0.37 for interval). Nevertheless, their potential influence remains physiologically and methodologically credible.

Regarding dose (cycles, cuff pressure, limb occluded mass): The typical RIPC scheme used in exercise science (3 or 4 cycles of 5 min occlusion at 170–220 mmHg) is derived from cardioprotection research [42, 45]. Theoretically, increasing stimulus magnitude (more cycles, higher pressure, larger limb volume) could enhance humoral and neural signalling, but the literature does not support a simple linear dose-response in acute performance contexts [45]. For example, Niespodzinski et al. (2021) found no additional benefit of increasing RIPC dose in a repeated-day protocol [17]. In our analysis, the 4 × 5-min subgroup did show a nominally higher pooled effect (SMD = 0.38) compared to 3 × 5-min (SMD = 0.10), but confidence intervals overlapped and heterogeneity remained high (I²=68%). This suggests that dose effects may exist but are moderated by other factors (participant training status, exercise modality, limb occlusion site) and require larger trials to detect reliably.

Concerning timing (interval between RIPC and exercise): The biphasic mechanistic model posits distinct early and late windows of effect [10, 33], whereby exercise performed too early may precede full mediator release, and too late may miss the acute benefits. However, our meta analysis did not find a statistically significant advantage for the proposed early window (< 30 min) compared to a later time point (≥ 30 min). Our data showed that the magnitude of the effect size was numerically larger for the < 30 min interval (SMD = 0.34) versus the ≥ 30 min interval (SMD = 0.10), though not statistically different and marked by substantial heterogeneity. Therefore, while the numerical difference in effect sizes aligns with the theoretical early window, our findings challenge the necessity of a strict sub-30-minute window for upper-body strength tasks. Future studies should therefore standardize and explicitly report the testing interval to clarify its role.

Additional moderators likely exist: participant characteristics (training level, sex, muscle mass of tested limb), exercise mode (1RM vs. repeated sprint vs. endurance), cuff location (arm vs. thigh), warm-up protocols, placebo/sham control, and outcome metrics (absolute vs. percentage changes) all influence responsiveness to RIPC. For instance, moderately trained individuals appear to benefit more than highly trained athletes, perhaps due to ceiling effects. The high between-study heterogeneity observed in prior reviews (I² often > 50% or 70%) underscores this complexity [46].

Notably, the observed effects in the strength endurance domain—which appeared to drive the nominally larger point estimates in some subgroups—were themselves characterized by substantial heterogeneity and a lack of statistical significance. This fundamental uncertainty limits the confidence in any conclusions regarding apparent moderator effects for RIPC on upper limb strength.

In conclusion, although our subgroup analyses did not reveal statistically significant moderator effects, the physiological plausibility and methodological variability suggest that number of cycles and interval timing remain key design variables that warrant rigorous investigation.

### Limitations of the study

This meta-analysis is constrained by the limited and heterogeneous evidence base currently available. Only a small number of trials (six randomized crossover studies) with a combined total of 84 healthy young men were found, which limits statistical power and generalizability. The modest sample sizes and diversity of study designs mean that our pooled estimates have wide confidence intervals and should be interpreted cautiously. Furthermore, for one study [34], the missing data necessitated extraction from figures using WebPlotDigitizer after unsuccessful attempts to contact the authors, which may introduce a potential source of measurement error. In fact, recent systematic reviews note that the certainty of evidence in this field tends to be moderate-to-low [22]. Many potential moderators (such as athletes’ training status, limb occlusion site, or warm-up procedures) vary across studies and could not be fully controlled. As O’Brien and Jacobs (2021) observe, “heterogeneous methods confound the comparison of results among various studies” in RIPC research [47]. For example, the number of ischemic cycles, cuff pressure, and timing of strength tests differed across experiments, making direct comparison difficult.

The characteristics of included studies also constrain generalizability. All trials enrolled young, healthy male adults, so it is unclear whether RIPC would have similar effects in women, older individuals, or clinical populations. The intervention protocols varied widely (cuff pressure 170–220 mmHg, 3 × 5 vs. 4 × 5 min cycles, upper-arm vs. thigh application, and different warm-up procedures) and the strength outcomes were heterogeneous (1RM, peak/mean power, repetitions to fatigue, etc.). This lack of standardization introduces uncontrolled confounding and makes it hard to isolate the true effect of RIPC. In short, methodological diversity (both in RIPC dosing and outcome measures) amplifies the risk that the pooled estimate is biased or not broadly applicable. Likewise, the crossover designs used in all trials also carry risks of carryover or learning effects, and blinding is challenging in RIPC protocols. Indeed, recent meta-analyses have highlighted that cognitive/placebo factors can strongly influence RIPC outcomes [22]. Taken together, these factors may obscure true effects.

Finally, because RIPC is an emerging field in sports science, our analysis may be affected by publication and reporting bias. As O’Brien and Jacobs (2021) note, journals may preferentially publish positive findings, potentially “obfuscating” null results [47]. In our review, funnel plots did not suggest major asymmetry, but with so few studies the risk of unpublished negative trials remains. In sum, while our work represents the first meta-analytic synthesis of RIPC on upper limb strength, the conclusions are constrained by the small, heterogeneous dataset. Future studies should address these gaps with larger samples, standardized methods, and broader participant groups.

### Practical implications and future directions of the study

For sports scientists and practitioners, our findings suggest that acute RIPC should be applied with caution as a strength-enhancing intervention. To date, evidence for meaningful improvement in upper-body strength is lacking, and coaches should not solely rely on RIPC as a guaranteed ergogenic aid. However, RIPC remains a low-cost, noninvasive technique that can be easily integrated into pre-exercise routines, and it may offer subtle benefits such as reduced fatigue or enhanced recovery in specific contexts. Given its safety and simplicity, practitioners who wish to experiment with RIPC may adopt standardized protocols—such as applying at least three 5-minute ischemia–reperfusion cycles bilaterally, as recommended by previous research [47]. Importantly, practical applications should be closely monitored, as placebo or expectation effects may partly explain perceived performance gains [46].

For researchers, several key directions arise from this meta-analysis. Larger, well-controlled randomized trials are needed to determine whether specific RIPC protocols produce consistent performance gains. Future studies should systematically vary intervention parameters—occlusion pressure (e.g., 170 vs. 220 mmHg), number of cycles, limb site, and timing between RIPC and exercise—to identify optimal conditions. For instance, our data showed a non-significant trend toward higher effects when exercise occurred within 30 min post-RIPC, suggesting that the “early window” may be more relevant than later testing. Trials should also include both sexes and diverse training backgrounds to improve generalizability, and explore chronic or repeated RIPC interventions integrated within training programs. Moreover, mechanistic studies employing physiological and biochemical markers (e.g., muscle oxygenation via NIRS, blood flow, oxidative stress indicators) will help clarify inter-individual variability in responsiveness. Finally, future meta-analyses could incorporate individual participant data and unpublished studies to enhance robustness and reduce bias. Collectively, these steps will refine RIPC research methodology and clarify whether this technique holds genuine value for sports performance or rehabilitation.

## Conclusion

This meta-analysis represents the first quantitative synthesis specifically examining the effects of remote ischemic preconditioning (RIPC) on upper-limb strength in healthy men. Across all included trials, pooled estimates showed a non-significant tendency favoring RIPC but failed to reach statistical significance for maximal strength, explosive strength, or strength endurance. While a moderate effect size was observed for strength endurance, the lack of statistical significance (*p* = 0.054) and substantial heterogeneity preclude any definitive conclusions about RIPC’s efficacy for this specific strength quality. These findings indicate that, under current experimental conditions, acute RIPC protocols do not consistently enhance upper-body performance. The limited number and heterogeneity of available studies further constrain the certainty of these conclusions, suggesting that apparent benefits may be influenced by methodological variation or placebo effects.

Nevertheless, this study provides a necessary evidence-based foundation for future work. By quantifying existing results and identifying methodological inconsistencies, it clarifies the current state of RIPC research and delineates directions for improvement. Theoretically, RIPC remains a promising intervention due to its potential roles in improving muscle oxygenation, neural adaptation, and fatigue resistance, but its practical efficacy in strength enhancement remains unproven. Moving forward, large-scale, standardized, and sex-inclusive trials are needed to determine whether specific RIPC protocols or timing strategies can reliably elicit performance gains. Until such evidence emerges, coaches and athletes should view RIPC as an experimental, low-risk approach rather than a confirmed ergogenic aid.

## Supplementary Information

Below is the link to the electronic supplementary material.

Supplementary Material 1

## Data Availability

The original contributions presented in the study are included in the article, further inquiries can be directed to the corresponding authors.
